# A Communication Partner Training Program Delivered via Telehealth for People Living With Parkinson's (Better Conversations With Parkinson's): Protocol for a Feasibility Study

**DOI:** 10.2196/41416

**Published:** 2023-02-03

**Authors:** Philippa Clay, Suzanne Beeke, Anna Volkmer, Lynn Dangerfield, Steven Bloch

**Affiliations:** 1 Division of Psychology and Language Sciences University College London London United Kingdom; 2 Adult Community Speech and Language Therapy Solent NHS Trust Portsmouth United Kingdom

**Keywords:** Parkinson’s, conversation, speech and language therapy, communication partner training, speech therapy, language therapy, communication difficulty, conversation skills

## Abstract

**Background:**

Parkinson’s can impact people’s speech, cognition, pragmatics, and language, significantly affecting their conversations with others. The speech and language therapy approach called communication partner training (CPT) is effective for a range of communication difficulties. However, speech and language therapy interventions for people with Parkinson’s predominantly focus on impairments, with little provision of CPT for this population. Better Conversations is a CPT approach that involves working with a dyad (the person with the communication difficulty and a conversation partner [CP]) to build conversation skills. It is effective at reducing barriers to conversation, and for some, it significantly increases targeted facilitatory strategies. Some approaches to CPT have been adapted to be delivered via telehealth. This can maximize ecological validity and convenience. Furthermore, telehealth is widely accepted as a delivery method for other interventions for Parkinson’s. This study presents the protocol for a pilot feasibility study of a Better Conversations CPT delivered via telehealth to people living with Parkinson’s and their CPs, called Better Conversations with Parkinson’s (BCP).

**Objective:**

The primary aim is to evaluate the feasibility of the BCP program delivered via telehealth with a treatment group from a collaborating National Health Service (NHS) site to establish for a main trial whether BCP can be delivered as intended in an NHS setting. The aim is to establish: (1) the acceptability of the program for people living with Parkinson’s, family members, and speech and language therapists (SLTs); (2) the feasibility of delivering the BCP program; (3) the recruitment and retention rates; (4) a sample size calculation; and (5) the most appropriate primary outcome measure.

**Methods:**

Ethical approval for this study was obtained from London-Central Research Ethics Committee (reference: 22/LO/0332). This case-series feasibility pilot study will recruit 10-12 dyads to ensure 10 complete data sets. Participants will be recruited by a collaborating NHS site located in England. Participants will be involved for 16 weeks (weeks 1-2 preintervention measures, weeks 3-8 intervention, weeks 10-12 postintervention measures, week 16 follow-up interview). Quantitative and qualitative methods will be used to analyze the study data. Speech, communication, and quality of life assessment data will be analyzed statistically to determine a suitably sensitive outcome measure. Descriptive statistics will be used to report on recruitment, attendance, and attrition. Finally, acceptability and feasibility will be evaluated using participant feedback, interviews, and the reflective diary and feedback of the SLT administering the therapy (by the research assistant who is the first author). This data will be analyzed using descriptive statistics and reflexive thematic analysis.

**Results:**

This study was approved for funding from Parkinson’s UK. Study recruitment commenced in July 2022. The results of the data analysis are expected to be available by September 2024.

**Conclusions:**

Insights from this study will provide valuable information about the acceptability and feasibility of a remotely delivered Better Conversations CPT approach for people living with Parkinson’s and their CPs. An outcome of this study will be a manualized BCP program coproduced by people living with Parkinson’s, their families, and a group of expert SLTs. The study results will guide the next stages of intervention development.

**International Registered Report Identifier (IRRID):**

PRR1-10.2196/41416

## Introduction

### The Impact of Parkinson’s on Speech and Communication

Parkinson’s is one of the fastest-growing neurological conditions in the world [1], with a recorded UK prevalence of around 22:10,000 women and 32:10,000 men [2]. Parkinson’s is associated with a loss of nerve cells in the substantia nigra, resulting in reduced dopamine. Changes to articulation, prosody, respiration, and voice are well attested. Disruption to communication can also occur because of changes in cognition, language, and pragmatics. Self-perception ratings suggest that around 90% of those living with Parkinson’s experience some speech-language changes [3,4], with speech among the top 4 concerns for 38% [3].

### Current Speech and Language Therapy Provision

Speech and language therapy interventions for people living with Parkinson’s have an established focus on intelligibility and volume and principally address speech impairment [5]. Intervention therefore typically focuses on approaches such as Lee Silverman Voice Treatment [6], which involves teaching people with Parkinson’s to recalibrate their volume through intensive speech exercises and can result in substantial improvements to vocal loudness and intelligibility [7]. A total of 85% of people with Parkinson’s who have had speech and language therapy feel that it has had a positive effect on communication and swallowing [4]. Those for whom speech and language therapy is not helpful cite reasons such as difficulty maintaining gains in day-to-day conversations; difficulty continuing exercises at home; and that therapy does not tackle all the aspects of communication relevant to them. Yorkston et al [8] similarly report difficulties maintaining therapeutic gains, and that therapy does not always address communication problems of concern, such as conversation and cognition. Suggestions for improvements to speech and language therapy include increased focus on the cognitive demands of speaking and psychosocial aspects of communication [9]. These perspectives are also reflected in the views of speech and language therapists (SLTs), who report feeling less able to address maintenance of therapeutic gains outside of therapy sessions [5] and describe lacking the necessary tools to target interaction or conversation in assessment and intervention with this client group [10].

### Communication Partner Training

Communication partner training (CPT) is an umbrella term for a therapy approach that involves working with a person with a communication difficulty and their conversation partner (CP; eg, a health professional, a family member, or a friend they regularly converse with) to improve everyday conversations. CPT is effective for a range of communication difficulties, such as aphasia [11], traumatic brain injury [12], and dementia [13]. Evidence suggests that CPT is effective at improving the knowledge, communication skills, and attitudes of communication partners [11,14-16]. Furthermore, CPT can enhance functional communication, accessibility, and participation for people with communication difficulties [11,16,17]. Common components of CPT programs across communication difficulties include the provision of information; building knowledge (eg, about strategies to enhance communication and negative behaviors to avoid), developing behavioral skills (the practical actions of participants in learning and practicing skills), and skill-building techniques [18].

### CPT for People Living With Parkinson’s

Despite similarities across CPT programs, individual strategies vary for different etiologies of communication difficulty [18]. Thilakaratne et al [19] carried out a scoping review aiming to describe the assessment methods and interventions used to treat conversations between people with Parkinson’s and their partners and to identify gaps in the literature. The authors found only 1 intervention study for people with Parkinson’s, which treated conversations by implementing CPT. This multiple case study [20] with 3 dyads suggests that CPT adapted for people living with Parkinson’s was well received and that the therapy may work well, although its effectiveness remains to be proven. Thilaraktne et al’s [19] scoping review highlights a need for further research implementing CPT for people living with Parkinson’s and incorporating a participation-based approach to intervention that involves all communication partners.

### The Better Conversations Approach to CPT

Better Conversations is an approach to CPT that aims to help people with communication difficulties have more enjoyable and successful interactions in their everyday lives [21]. Rather than working with solely the person with communication difficulties or their communication partner, it involves both parties, referred to as a dyad, in building conversation skills. The SLT supports a dyad to video-record themselves in natural conversations and uses clips from the videos to help them identify what is working well in conversation (facilitators) and what gets in the way (barriers). The dyad sets individualized personal goals and is supported in practicing chosen strategies in order to build their skills in conversation. Behavior change techniques are targeted at both members of the dyad. The most established Better Conversations program is Better Conversations with Aphasia [22], which significantly reduces barriers to conversation at the group level and, for some individuals, significantly increases targeted facilitatory strategies [23]. Previous successful adaptations of Better Conversations include the coproduced Better Conversations with Primary Progressive Aphasia [13]. This has been deemed acceptable and feasible to deliver, and initial pilot data demonstrates promise as a means to improve self-identified communication behaviors between members of the dyad [24].

### Telehealth

Telehealth or telemedicine, the “delivery of health care services where distance is a critical factor” [25], can improve access to quality, cost-effective health services. Despite barriers, in particular for those with cognitive and visual impairments [26], telehealth offers advantages, such as removing issues related to cost, travel, and inconvenience for the service user; increasing access to health care; and being able to observe people in their own environment [27,28]. Better Conversations, and CPT in general, has been adapted to a telehealth form of delivery. Beeke et al [29] introduced the term “teleCPT” to mean CPT delivered via videoconferencing technology. In this case study of a person living with primary progressive aphasia and his partner, the authors demonstrate that Better Conversations delivered via videoconferencing is effective, acceptable, and feasible to deliver. Furthermore, teleCPT can maximize the ecological validity and convenience of therapy [29]. Theodoros et al [30] suggest that telerehabilitation has particular potential to improve services for people with Parkinson’s: it allows ready access to treatment, it supports the development of self-management skills, and people with Parkinson’s express overall satisfaction with and support for telerehabilitation. There is, however, an absence of evidence exploring interaction-focused therapies or CPT delivered via telehealth (teleCPT) to people living with Parkinson’s.

### Summary

There is demand from people living with Parkinson’s for speech and language therapy interventions that address broader aspects of communication beyond speech, involve family members, and consider psychosocial factors. However, there is limited evidence for CPT among people living with Parkinson’s. CPT has been effective at improving communication participation for people living with other communication difficulties, and initial research suggests it is acceptable and may be beneficial for people living with Parkinson’s [31]. This study presents the protocol for a pilot feasibility study of a Better Conversations CPT for people living with Parkinson’s and their CPs, called Better Conversations with Parkinson’s (BCP). Telehealth has been chosen as the primary method of delivery for this program due to the high levels of reported acceptability among this client group and its ability to increase access to therapy and support people within their everyday environment.

This feasibility study has been designed in line with Medical Research Council Guidance on developing complex interventions [32]. BCP has been under development through adaptation of existing Better Conversations interventions, identification of key mechanisms of behavioral change, and progressive refinement of the design through coproduction work. The program has been coproduced with people living with Parkinson’s, family members, and expert SLTs. A steering group (of people living with Parkinson’s, family members, and expert SLTs) was established to provide advice and feedback on all aspects of study management. This study addresses the feasibility phase of development by considering the evaluation design (recruitment, outcomes, and analysis) and the intervention (optimal content and delivery, acceptability).

### Aim

The primary aim of this study is to evaluate the feasibility of the BCP program delivered via telehealth with a treatment group from a collaborating National Health Service (NHS) site to establish for a main trial whether BCP can be delivered as intended in an NHS setting.

Specifically, the aims are to establish (1) the acceptability of the program for people living with Parkinson’s, family members, and SLTs; (2) the feasibility of delivering the BCP program; (3) the recruitment and retention rates; (4) a sample size calculation; and (5) the most appropriate primary outcome measure.

## Methods

### Design

**Figure 1:**
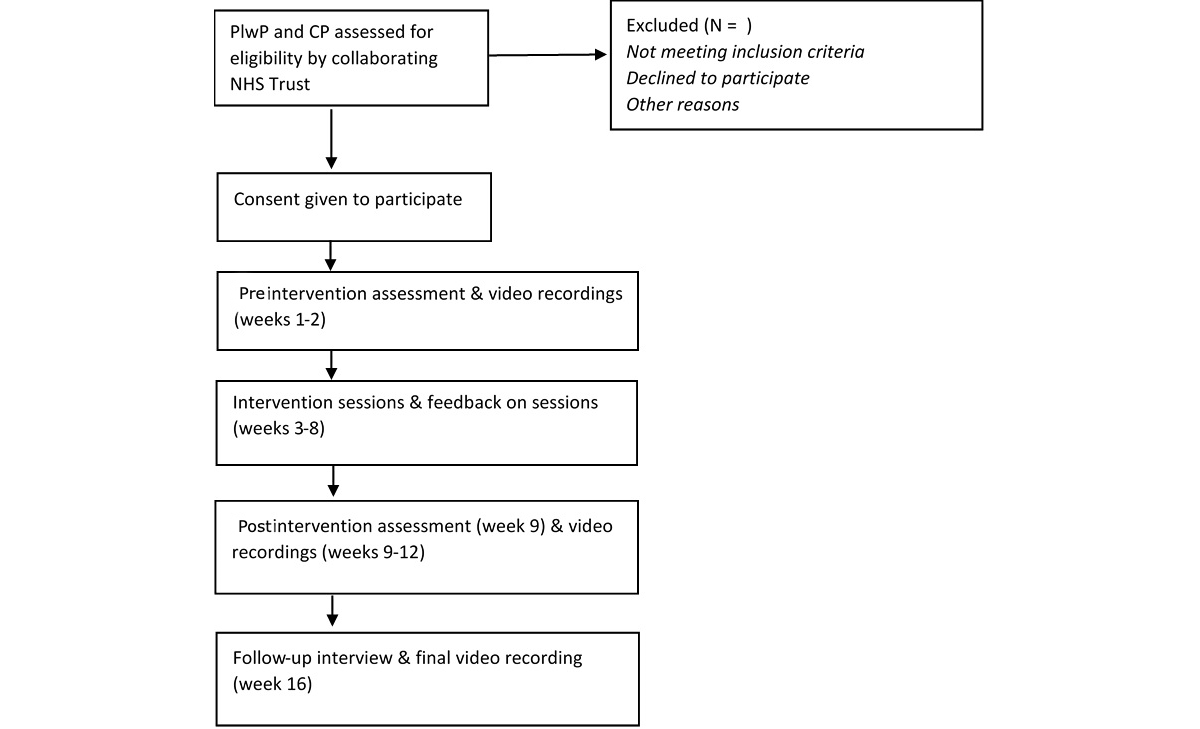
Participant flow through the study. CP: conversation partner; PlwP: persons living with Parkinson’s.

This is a case-series feasibility pilot study of the BCP therapy program. Participants will be involved for 16 weeks: preintervention measures (weeks 1-2), intervention (weeks 3-8), postintervention measures (weeks 9-12), and a follow-up interview 4 weeks later (week 16). Figure 1 shows participant flow through the study.

### Setting

Participants will be recruited by SLTs at a collaborating NHS site located in England. Consent, preintervention measures, and delivery of the BCP intervention will be carried out by a research assistant (a specialist SLT by background; the first author) employed by the study sponsor. The collection of outcome measures and delivery of therapy will be done remotely (ie, via a computer, tablet, or smartphone). Participants will be offered a tablet device to take part in the therapy if required, although an existing internet connection will be required.

### Population

The study includes adults (>18 years) with a diagnosis of Parkinson’s and related communication difficulties and a chosen CP. Local collaborators (SLTs) at participating NHS sites will use medical notes and a case history to judge potential participants against the inclusion and exclusion criteria (Textbox 1).

**Participant inclusion and exclusion criteria.**

**Inclusion criteria**

1. Being 18 years or older
2. Having a diagnosis of Parkinson’s
3. Experiencing intelligibility or communication problems relating to Parkinson’s
4. Having some ability to communicate and understand communication in order to participate in the Better Conversations with Parkinson’s (BCP) program
5. Being able to see and hear well enough to participate in the program
6. Being functionally able to engage in the program (ie, able to maintain some concentration and remain in a 60-90 minute session; minimal challenging behavior so as to be unlikely to cause disruption)
7. Having English as their language of daily use
8. Having a conversation partner (CP) who is able to and consents to participating in the project
9. Being able and ready to actively participate in conversation therapy at the current time

**Exclusion criteria**

1. Having a history of brain lesions or major head trauma
2. Having a major physical illness or disability that could impact participation
3. Present with a major psychiatric diagnosis
4. Present with prominent behavioral disturbances
5. Present with prominent episodic memory, visual memory, or visuo-perceptual impairments
6. Currently participating in active speech and language therapist (SLT) intervention that may conflict with BCP
7. Participating in or having recently participated in research which may conflict with BCP
8. Having no access to a Wi-Fi connection in order to be able to access BCP remotely

### Identification and Recruitment

SLTs at a collaborating NHS Trust will be asked to identify potential participants using the inclusion and exclusion criteria (Textbox 1) and invite them to participate via a flyer. People who meet the inclusion criteria will not be under any obligation to take part in this study, and this will be made clear from the outset. Those who express interest will be given the participant information sheet (PIS) and sent an electronic link. Potential participants can use the link to indicate their consent to being contacted by the research team and to securely share their contact details. Potential participants will be contacted for consent to participate in the study at least 48 hours after receiving the PIS and sharing contact details.

Local collaborators will complete a log to record the number of people with a diagnosis of Parkinson’s who do not meet the inclusion criteria and the number of people who are eligible but decline to participate, with their reasons why if provided. Participants will be reviewed by the local collaborator SLT after finishing the study. At this point, participants will resume routine speech and language therapy with the collaborating NHS Trust.

### Ethics Approval

**Figure 2:**
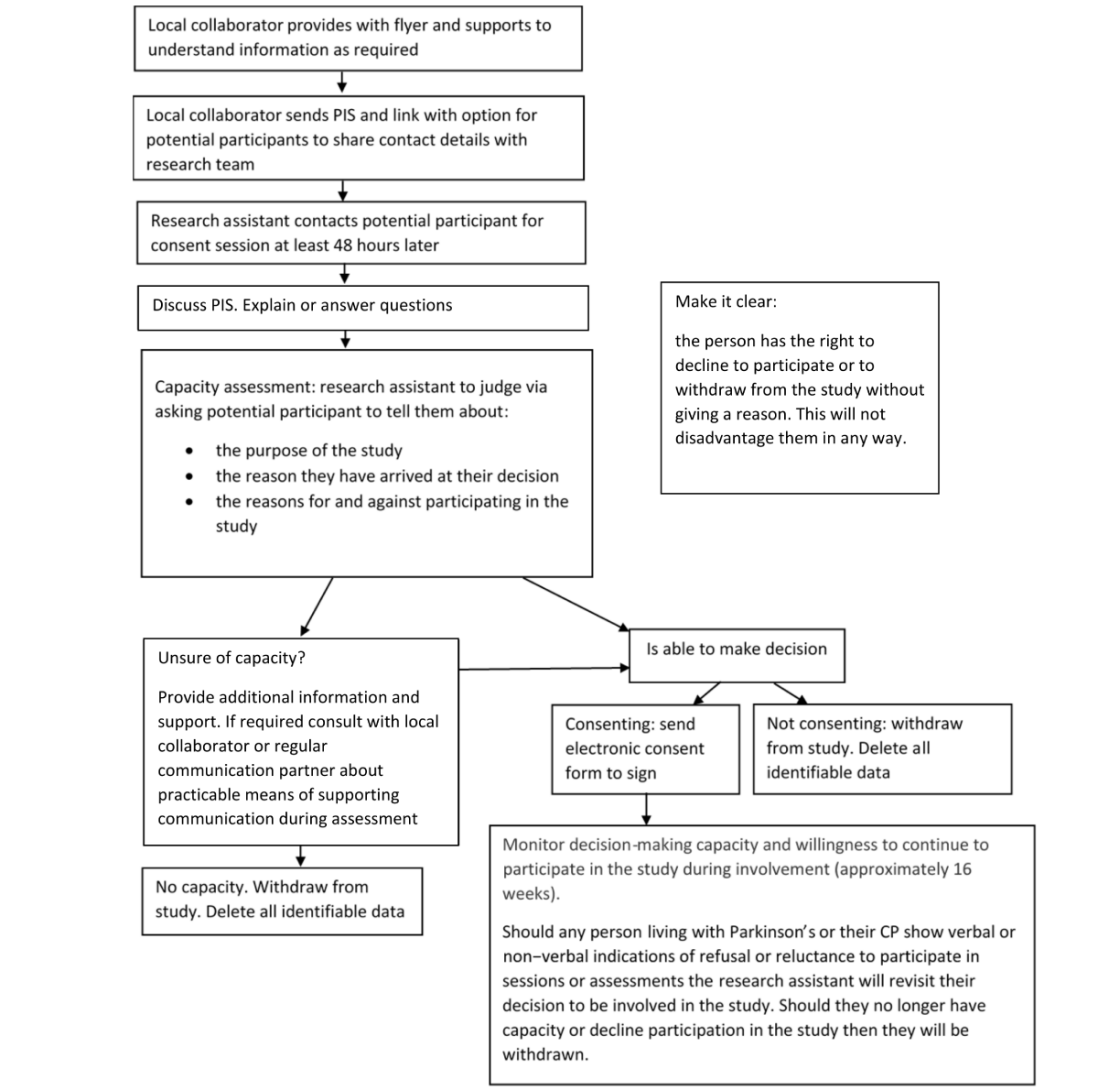
Process of consent. CP: conversation partner; PIS: participant information sheet.

Ethical approval was granted by the London-Central Research Ethics Committee (reference 22/LO/0332). Figure 2 depicts the process of consent. Participants in the study are expected to be in the mild to moderate stages of Parkinson’s and therefore likely to be competent to give informed consent to participate, provided that sufficient time is allowed for them to reach a decision. Those who are unable to give informed consent will not be able to take part in the study (see Figure 2). Informed consent will be obtained by the research assistant following the current guidance from the Mental Capacity Act [33], Royal College of Speech and Language Therapists guidelines [34], and Good Clinical Practice Standards [35]. The research assistant is a specialist SLT with considerable experience supporting people with communication impairments and issues related to obtaining consent. Potential participants will be given specialist support to understand the information sheets by the research assistant as required.

Participants will not be financially compensated for taking part in this study. All participant data will be deidentified where possible. Identifiable information will remain confidential and will be stored securely in line with the Data Protection Act 2018. See below for further information about data management.

### Sample Size Justification

The recruitment of participants will be dealt with pragmatically, within the scope of a small feasibility pilot study. Based on related research and discussions with clinicians at the collaborating NHS Trust, who estimate an active caseload of people living with Parkinson’s of approximately 90 cases (inclusive of swallowing and communication difficulties), it is judged that it will be possible to identify and recruit 10-12 couples over a 6-month period. This allows for a slight overrecruitment to ensure 10 complete data sets.

### Outcome Measures

Participants will complete preintervention speech, intelligibility, functional communication, and quality of life measures (Multimedia Appendix 1) with the research assistant in weeks 1-2 of the study. Questionnaires will be delivered via a mutually accessible platform (eg, Microsoft Teams or Zoom) or through an electronic questionnaire depending on participant choice, as recommended by the research steering group. Participants will be interviewed to establish a profile of their typical interactions [36], the types of problems with interaction they experience, how often these occur, and in what situations they arise. This will include the perspectives of the person living with Parkinson’s and their CP. They will also complete a case history, a rating scale of Parkinson’s symptoms, and a cognition and speech assessment to allow a description of relevant individual participant characteristics. This preintervention assessment protocol mirrors routine speech and language therapy practice. Furthermore, it provides the opportunity for the research assistant to build rapport with participants before delivering the intervention.

After intervention, measures (Multimedia Appendix 1) will be repeated (weeks 9-12). These assessments will contribute to the identification of a primary outcome measure for a future trial, if this is warranted. Final follow-up interviews and the Better Conversations Self Rating Scale will take place 8 weeks after treatment to understand long-term acceptance and the impact of the intervention. This will include the perspectives of both the person living with Parkinson’s and their CP. Repeated measures and the follow-up interview will be conducted by junior researchers (student SLTs) with training and support from the chief investigator and research assistant.

Each dyad will be supported to video-record themselves in natural conversation for 15 minutes, 3 times before and 3 times after intervention. They will be recorded via a laptop or tablet by a member of the research team using a mutually accessible platform (eg, Zoom or Microsoft Teams). The team member will set up the remote meeting, start the recording, and then turn off their camera and microphone to leave the dyad to talk unobserved. If the dyad requires it, a list of conversation topics will be provided to support this process. This form of remote data collection replicates methods used in previous teleCPT and Better Conversations research [29] and is designed to facilitate ease of data collection and security of data transfer. The timescales of sample video recordings are shown in Table 1.

**Table 1:** Schedule of conversation sample recordings.

	Consent session	Preintervention assessment	BCP^a^ intervention (6 weeks)	Postintervention assessment	Final data collection	Follow-up interviews or rating scales
Week (expected timescale)	0	1-2	3 -8	9	10-12	16
Conversation sample recording	N/A^b^	1, 2, and 3	N/A	4	5 and 6	7

^a^BCP: Better Conversations with Parkinson’s.

^b^N/A: not applicable.

### Intervention

The BCP program is described in Multimedia Appendix 2, using the Template for Intervention Description and Replication (TIDieR) checklist. BCP is based on the principles of a Better Conversations approach to CPT [21] and is the first of its kind to meet the specific needs of people living with Parkinson’s. It has been coproduced by people living with Parkinson’s and SLTs who are experts in this area. Participants’ preintervention video-recorded conversation samples will be used to provide short clips for video feedback during intervention sessions. Each member of the dyad will identify personal goals depending on their conversation challenges, but each session’s objectives, activities, and types of feedback are manualized. The approach is underpinned by the COM-B model of behavior change [37] and is designed to target behavior change techniques from the Behavior Change Techniques Taxonomy [38], in particular those reliably identified as active ingredients in Better Conversations with Aphasia [39]. The BCP intervention will be administered by the research assistant.

### Evaluation of Acceptability

The acceptability of the intervention and study procedures will be evaluated qualitatively. Online anonymous questionnaires submitted after each therapy session will obtain the participants’ views and experiences of session delivery, objectives, and activities. Semistructured interviews will be conducted by junior researchers 8 weeks postintervention to explore acceptability, including participant attitude toward and experience of the intervention; the impact of the intervention on activity, participation, and well-being; what participants perceive to be the barriers and enablers to implementing strategies from the intervention; what participants valued about the intervention; and what changes participants might suggest to the intervention. Additionally, the research assistant administering the intervention will complete a reflective diary and feedback questionnaires on the experience of delivering therapy, whether sessions met expectations, adaptations required to sessions, and the perceived helpfulness of therapy for the dyad. This data will be analyzed by junior researchers to enhance objectivity.

### Evaluation of Feasibility

Feasibility will be evaluated by collecting data on the following: recruitment and retention rates; time required to recruit to target; attrition rates; feasibility of pre- and postintervention assessment and data collection methods, including completion rates; adverse events.

### Data Management

The study is compliant with the Data Protection Act 2018. All personal information (contact details, date of diagnosis, medical and social history) will remain confidential. Each participant will be given a unique research number, which will be used on all paperwork, in the names of video files and electronic records (eg, assessment data), and in analysis documents and subsequent publications. All data will be stored securely on Data Safe Haven, in accordance with the Data Protection Act 2018 and the sponsor’s data protection policy.

Video recording of conversation samples between dyads is required as an outcome measure and for video feedback during the intervention. Participants will consent to being video recorded for these purposes. Faces are required to be visible for detailed analysis of the video recordings, as natural human communication is the focus of the intervention being piloted. Only the research team will have access to the full video-recorded data set. Participants will be given the choice to opt in or out of the additional use of short extracts of video recordings for presentations about the research to professional audiences, teaching purposes, and as part of an online therapy resource. It is made clear in the PIS that there are risks of being recognized, and participants will be given a choice as to whether videos are masked (voice and image altered to reduce recognizability) if used for these purposes. They can opt out of videos being used in this way at any point during the study.

No data management committee will be established as it is felt that this short, small-scale pilot carries minimal risks. If any information is disclosed by the participant that leads the chief investigator to believe that a participant is at risk of harm or harming others, then confidentiality will be broken to ensure the safety of the person or people involved.

### Data Analysis

Quantitative and qualitative methods will be used to analyze the study data. Descriptive statistics will be used to report on recruitment, attendance, and attrition. Recruitment and retention rates will be used to support sample size calculations to inform a future full trial and plan the required number of sites to meet this target. To inform a sample size calculation, the mean pre- and postintervention scores, a mean change score, 95% CIs, and SDs will be calculated for each outcome measure. The mean change scores and standard deviations will be entered into the G*Power software, and an effect size will be calculated; this will then inform a 2-tailed sample size calculation for each measure.

Recordings of adverse events and participant feedback will be used to inform future recruitment procedures. The analysis of acceptability data will be based on participant feedback forms, interviews, and adherence data. This data will be analyzed using descriptive statistics and reflexive thematic analysis [40].

Speech, communication, and quality of life assessment data will be entered into a database and analyzed using SPSS (IBM Corp). This information will be used to determine a suitably sensitive outcome measure for a next-stage clinical trial, should this be warranted. Conversation outcomes will be captured using a procedure developed for Better Conversations with Aphasia to identify changes in the use of targeted strategies following intervention [23]. This will involve analysis of frequency counts of barrier and facilitator behaviors linked to people’s personal goals for intervention in 5-minute video samples selected from the midpoint of each of 6 pre- and postintervention conversation samples. We hypothesize that dyad management of problems with intelligibility (repair) may be a key site of conversation change. Intelligibility problems typically involve both speakers in a dyad over a series of conversational turns [41]. Given this, a secondary evaluation of conversation data will be conducted using Conversation Analysis [42].

### Criteria for Success

The study will be considered appropriate for a next-stage clinical trial evaluating the intervention if (1) the intervention is acceptable to people with Parkinson’s and their CP, (2) the intervention is feasible to deliver for the SLT, (3) a suitable sensitive outcome measure is identified, and (4) an appropriate sample size is estimated.

### Assessment and Management of Risk

This is a low-risk study. There is a possibility that participants will not experience improvements in their communication, well-being, or quality of life as a result of the intervention. However, there is no evidence to indicate that participants will experience harmful side effects, and the intervention has been coproduced with people living with Parkinson’s and expert SLTs to maximize acceptability and relevance. There is evidence to suggest that this kind of intervention is effective at improving communication for adults with other communication difficulties and their partners.

Most assessments and procedures, such as videoing, are routinely used in speech and language therapy clinical practice. It is possible that distress may arise from discussions of communication difficulties and from measures or therapy tasks highlighting existing problems in conversation. The research assistant carrying out the intervention is a highly trained SLT with the ability to manage sensitive situations. The risk of distress is expected to be balanced by the opportunity to discuss communication strengths and strategies, as well as the enjoyment in targeted conversation time with a nominated partner. The process of being recorded on video and watching yourself on video can be confronting. The coproduction group (including people living with Parkinson’s, their family members, and specialist SLTs) advised on how to introduce video to participants, so that as far as possible, participants are familiarized with the process and understand why video is vital to changing conversation behavior in a Better Conversations approach.

All study procedures are outlined in the PIS, which has been designed with support from people living with Parkinson’s and their partners. Participants will be reminded that they can withdraw from the study at any time. Any adverse events will be recorded in the participant’s medical record, and the study sponsor will be informed.

## Results

This study was approved for funding from Parkinson’s UK in December 2020 following peer review of the proposal by an independent committee (Multimedia Appendix 3). This study was approved for funding from Parkinson’s UK in December 2020. Study recruitment commenced in July 2022. The results of the data analysis are expected to be available by September 2024.

## Discussion

To the authors’ knowledge, this is the first study to investigate a remotely delivered CPT approach for people living with Parkinson’s. This study outlines the protocol for the study with the aim of evaluating the acceptability of the BCP program for people living with Parkinson’s, family members, and SLTs, and the feasibility of delivering the BCP program in collaboration with an NHS site. The study aims to identify a sample size calculation, recruitment and retention rates, and the most appropriate primary outcome measure to guide the future development of the BCP intervention.

This study involves the adaptation of the Better Conversations approach to CPT for a new client group. There is evidence that a Better Conversations approach reduces barriers to conversation and can increase the use of facilitatory strategies [23]. This study also builds on previous research by Forsgren et al [20] by further investigating a CPT approach for people living with Parkinson’s and, in addition, trialing teleCPT, therefore using a form of delivery that has been found to be highly acceptable for this client group [30]. Despite psychosocial and language issues being flagged as an important reason for referral for people living with Parkinson’s, the evidence base and SLT clinical practice are predominantly focused on swallowing, voice, and articulation [5]. This program has the potential to address psychosocial factors and communication difficulties beyond speech and, therefore, fill a gap in research and current clinical practice.

CPT has been shown among other client groups to enhance communication and participation [11] and result in changes to CPs’ knowledge, skills, and attitudes [14,15]. This study will provide valuable information about the sensitivity of measures in these areas for capturing CPT outcomes for people living with Parkinson’s and CPs. Additionally, this research will provide valuable information about the acceptability and feasibility of the Better Conversations approach for people living with Parkinson’s and their CPs. An outcome of this study will be a manualized BCP program coproduced by people living with Parkinson’s, their families, and a group of expert SLTs and adapted following the feedback of this study’s participants.

The outcomes of this study will be used to guide future phases in the development of the BCP intervention, as per Medical Research Council guidance for developing and evaluating complex interventions [32]. For example, given that this study investigates BCP delivered via telehealth, a future phase of research may compare face-to-face and telehealth delivery. A longer-term aim is to create a freely accessible intervention resource for the benefit of people living with Parkinson’s and SLTs working with them.

### Limitations

As a small-scale feasibility study with 10-12 dyads from 1 NHS site, the study is limited in scope. The study will therefore not seek to evaluate effectiveness or to generalize results beyond the participants. As a case-series study, which aims to describe the characteristics and outcomes at an individual level of a group of participants with Parkinson’s, this study does not include a control group. The results of the study will guide future phases of research into the BCP intervention, which if appropriate may involve a randomized controlled trial design.

A possible risk is a lack of diversity due to recruiting from 1 geographic area and an SLT caseload. Ascertainment bias is also possible: outcomes may be affected by factors (eg, socioeconomic status and cultural and linguistic barriers) that might be limiting access to the local speech and language therapy service. Despite the provision of a device to access the research, exclusion is possible as a result of digital poverty due to the requirement to have an internet connection to take part. Due to resource limitations, the research assistant will carry out the initial assessment and intervention. There is a risk that participants will not feel able to be completely open with feedback following each therapy session as the SLT delivering therapy is a member of the research team. The postintervention assessment and follow-up interviews will be completed by trained junior researchers to ensure that they are unaware of premeasure values and to provide an opportunity for participants to provide feedback they might not want to disclose to the SLT research assistant.

Personalization is a key element of this therapy approach. The coproduction group, which involves people with Parkinson’s and SLTs, has created a core set of activities and session plans to be used across all participants but has confirmed that personalization is essential. It is recognized that tailoring the intervention to different dyads and their goals may result in variation in the types of strategies identified and practiced; however, the session objectives, activities, and feedback tasks will be the same for all.

### Dissemination

The authors aim to disseminate the results of this study in peer-reviewed scientific journals and via presentations at academic and clinical conferences. Results will also be disseminated via user group publications and will be publicized via the BCP Twitter account and Better Conversations web pages. Opportunities to disseminate outcomes of the study to people living with Parkinson’s, SLTs, and researchers will be created through university and NHS Trust links and supported by the study steering group.

### Conclusions

This study will provide valuable information about the acceptability and feasibility of BCP—a CPT approach for people living with Parkinson’s and their CPs—delivered via telehealth. Outcomes of this study will include a manualized BCP program coproduced by people living with Parkinson’s, their families, and expert SLTs, which will be refined based on feedback from study participants. The study results will guide the next phases of intervention development, with the long-term aim of creating a freely accessible intervention resource for SLTs to use with people living with Parkinson’s and their regular CPs.

## Appendix

Multimedia Appendix 1 Outcome measures and method of collection.

Multimedia Appendix 2 Template for Intervention Description and Replication (TIDieR) Checklist.

Multimedia Appendix 3 Peer-review reports on grant proposal.

## Abbreviations

BCP: Better Conversations with Parkinson’s
CP: conversation partner
CPT: communication partner training
NHS: National Health Service
PIS: participant information sheet
SLT: speech and language therapist

## Data Availability

The data sets generated and analyzed during this study are available from the corresponding author on reasonable request, subject to consent.
