# Supplementary material for: A Communication Partner Training Program Delivered via Telehealth for People Living With Parkinson's (Better Conversations With Parkinson's): Protocol for a Feasibility Study
Source: JMIR Res Protoc. 2023 Feb 3;12:e41416. doi: 10.2196/41416 (PMC9938441; doi:10.2196/41416)
Supplement: Multimedia Appendix 1 [file resprot_v12i1e41416_app1.docx]

## Appendix 1: Outcome measures & method of collection

| **Measure** | **Method of collection** | **When collected** |
| --- | --- | --- |
| Biographical data (e.g. age, medical history, disease duration) and collection and profile of typical interactions | Interview via videoconference with research assistant | Pre-intervention only |
| The Frenchay Dysarthria Assessment-2 [1] | Person living with Parkinson’s (plwp) via videoconference with research assistant | Pre-intervention only |
| MDS-Unified Parkinson's Disease Rating Scale (MDS-UPDRS) [2] | Via videoconference with research assistant | Pre-intervention only |
| The Montreal Cognitive Assessment (MoCA) [3] | Via videoconference with research assistant | Pre-intervention only |
| The Voice Handicap Index [4] | Questionnaire for plwP  Support via videoconference as required | Pre- and post- intervention |
| Dysarthria Impact Profile [5] | Questionnaire for plwP  Support via videoconference as required | Pre- and post- intervention |
| Communicative Participation Item Bank [6] | Questionnaire for plwP  Support via videoconference as required | Pre- and post- intervention |
| Parkinson's Disease Questionnaire (PDQ-39) [7] | Questionnaire for plwP  Support via videoconference as required | Pre- and post- intervention |
| Parkinson’s Disease Questionnaire – Carers (PDQ-Carer) [8] | Questionnaire for conversation partner (CP)  Support via videoconference as required | Pre- and post- intervention |
| Conversation partner questionnaire investigating knowledge, skills and feelings around communicating with person with Parkinson’s | Questionnaire for CP  Support via videoconference as required | Pre- and post- intervention |
| Goal Attainment Scaling (GAS) in rehabilitation [9] | Plwp and CP via videoconference | Within and post- intervention |
| Better Conversations Self Rating Scales [10] as described in the Better conversations with Aphasia resource [11] | Plwp and CP via videoconference | Pre- and post- intervention and at 8 week follow up |
| Measure of Participation in Conversation & Measure of Skill in Supported Conversation [12] | Analysis of conversation sample videos by research team | Pre- and post- intervention |
| Better Conversations checklist of barriers and facilitators [13] as described by Best et al. [14] and in the Better Conversations with Aphasia resource [11] | Analysis of conversation sample videos by research team | Pre- and post- intervention |

1. Enderby P, Palmer R. Frenchay Dysarthria Assessment 2 Edition (FDA-2). Austin: Pro-Ed Inc; 2008.

2. Goetz CG, Tilley BC, Shaftman SR, Stebbins GT, Fahn S, Martinez-Martin P, et al. Movement Disorder Society-sponsored revision of the Unified Parkinson’s Disease Rating Scale (MDS-UPDRS): Scale presentation and clinimetric testing results. Mov Disord. John Wiley & Sons, Ltd; 2008;23(15):2129–2170.

3. Nasreddine ZS, Phillips NA, Bédirian V, Charbonneau S, Whitehead V, Collin I, et al. The Montreal Cognitive Assessment, MoCA: A Brief Screening Tool For Mild Cognitive Impairment. J Am Geriatr Soc. John Wiley & Sons, Ltd; 2005;53(4):695–699.

4. Jacobson BH, Johnson A, Cynthia G, Silbergleit A, Jacobson G, Benninger MS, et al. The Voice Handicap Index (VHI). Am J Speech-Language Pathol. American Speech-Language-Hearing Association; 1997;6(3):66–70.

5. Walshe M, Peach RK, Miller N. Dysarthria Impact Profile: development of a scale to measure psychosocial effects. Int J Lang Commun Disord. John Wiley & Sons, Ltd; 2009;44(5):693–715.

6. Baylor C, Yorkston K, Eadie T, Kim J, Chung H, Amtmann D. The Communicative Participation Item Bank (CPIB): item bank calibration and development of a disorder-generic short form. J Speech Lang Hear Res. 2013;56(4):1190–1208.

7. Jenkinson C, Fitzpatrick RAY, Peto VI V., Greenhall R, Hyman N. The Parkinson’s Disease Questionnaire (PDQ-39): development and validation of a Parkinson’s disease summary index score. Age Ageing. 1997;26(5):353–357.

8. Jenkinson C, Dummett S, Dawson J, Fitzpatrick R, Kelly L, Morley D, et al. PND57 A measure of carer quality of life in Parkinson’s Disease (PDQ-Carer): Development and validation of a summary index score. Value Heal. Elsevier; 2012;15(7):A556.

9. Turner-Stokes L. Goal attainment scaling (GAS) in rehabilitation: a practical guide. Clin Rehabil. SAGE Publications Ltd STM; 2009;23(4):362–370.

10. Johnson F. Better Conversations Rating Scales. Unpublished. University College London; 2011.

11. Beeke S, Sirman N, Beckley F, Maxim J, Edwards S, Swinburn K, et al. Better Conversations with Aphasia: an elearning resource [Internet]. 2013.

12. Kagan A, Winckel J, Black S, Felson Duchan J, Simmons-Mackie N, Square P. A set of observational measures for rating support and participation in conversation between adults with aphasia and their conversation partners. Top Stroke Rehabil. Taylor & Francis; 2004;11(1):67–83.

13. Beeke S, Beckley F, Best W, Edwards S, Johnson F, Maxim J, et al. Better Conversations Checklist of Facilitators and Barriers. Unpublished. University College London; 2013.

14. Best W, Maxim J, Heilemann C, Beckley F, Johnson F, Edwards SI, et al. Conversation therapy with people with aphasia and conversation partners using video feedback: A group and case series investigation of changes in interaction [Internet]. Front Hum Neurosci. 2016. p. 562.
