# Supplementary material for: A Communication Partner Training Program Delivered via Telehealth for People Living With Parkinson's (Better Conversations With Parkinson's): Protocol for a Feasibility Study
Source: JMIR Res Protoc. 2023 Feb 3;12:e41416. doi: 10.2196/41416 (PMC9938441; doi:10.2196/41416)
Supplement: Multimedia Appendix 3 [file resprot_v12i1e41416_app3.pdf]

Appendix 3: Peer review reports on grant proposal

As part of the grant funding process the study proposal was peer-reviewed by an independent committee including scientific experts in Parkinson's research and lay grant reviewers (people living with Parkinson's). The submitted protocol has taken into account reviewer comments. For example, the following changes were made to the protocol:

- clarification about the intervention in the summary and using the Template for Intervention Description and Replication (TIDieR) checklist
- including loan of iPads to those who require them, to reduce potential barriers for those with reduced technological access
- including an interview about how partners typically interact pre-intervention to understand how the video samples differ from everyday conversations
- including a pre-intervention clinical-neurological assessment of the participants to better understand the targeted population
- clarifying the role of the research assistant
